# Field-Induced Agglomerations of Polyethylene-Glycol-Functionalized Nanoclusters: Rheological Behaviour and Optical Microscopy

**DOI:** 10.3390/pharmaceutics15112612

**Published:** 2023-11-10

**Authors:** Sandor I. Bernad, Vlad Socoliuc, Izabell Craciunescu, Rodica Turcu, Elena S. Bernad

**Affiliations:** 1 Centre for Fundamental and Advanced Technical Research, Romanian Academy—Timisoara Branch, Mihai Viteazul Str. 24, RO-300223 Timisoara, Romania; vsocoliuc@gmail.com; 2 National Institute for Research and Development of Isotopic and Molecular Technologies (INCDTIM), Donat Str. 67-103, RO-400293 Cluj-Napoca, Romania; izabell.craciunescu@itim-cj.ro (I.C.); rodica.turcu14@gmail.com (R.T.); 3 Department of Obstetrics and Gynecology, Faculty of General Medicine, University of Medicine and Pharmacy “Victor Babes” Timisoara, P-ta Eftimie Murgu 2, RO-300041 Timisoara, Romania; ebernad@yahoo.com

**Keywords:** magnetoresponsive nanocomposite, particle aggregation/agglomeration, magnetic particle targeting, chain formation, magnetorheological properties, optical microscopy

## Abstract

This research aims to investigate the agglomeration processes of magnetoresponsive functionalized nanocluster suspensions in a magnetic field, as well as how these structures impact the behaviour of these suspensions in biomedical applications. The synthesis, shape, colloidal stability, and magnetic characteristics of PEG-functionalized nanoclusters are described in this paper. Experiments using TEM, XPS, dynamic light scattering (DLS), VSM, and optical microscopy were performed to study chain-like agglomeration production and its influence on colloidal behaviour in physiologically relevant suspensions. The applied magnetic field aligns the magnetic moments of the nanoclusters. It provides an attraction between neighbouring particles, resulting in the formation of chains, linear aggregates, or agglomerates of clusters aligned along the applied field direction. Optical microscopy has been used to observe the creation of these aligned linear formations. The design of chain-like structures can cause considerable changes in the characteristics of ferrofluids, ranging from rheological differences to colloidal stability changes.

## 1. Introduction

The potential for preventing thrombotic events and limiting post-angioplasty restenosis with rapid endothelium recovery and restoring its normal activities [1,2,3] justifies the development of techniques for quickening arterial re-endothelialization.

For the treatment of chronic diseases, localized therapies that use pharmaceuticals targeted by magnetic carriers are particularly appealing since they can get around the dose limit toxicity constraint while increasing drug efficacy [4]. Using a static magnetic field (SMF), magnetic guiding improves the deposition of drug carriers that are magnetically responsive when injected [5,6].

Magnetically guided medication delivery is impacted by three vital biophysical processes: (i) drug-loaded particle transport through the arteries, (ii) extravasation via blood vessel walls, and (iii) interstitial transport inside tissue.

It is crucial to note that when using magnetic drug targeting (MDT), medications are administered only by touching the artery wall. Magnetic nanoparticles (MNPs) are only captured from the blood flow when the following applies:

(a) They are in contact with the artery wall.
(b) They hold in place. In this situation, the magnetic force component must be vital enough to counteract the drag force and keep a particle in place.

Iron oxide particles (IONPs), particularly magnetite (Fe_3_O_4_) and maghemite (ɣ-Fe_2_O_3_), are the most widely employed magnetic materials among the many classes of MNPs, particularly in biomedical applications [7]. Their surface alterations, in addition to their inherent physicochemical characteristics, control this potential.

Numerous recent studies on the advantages of using magnetic iron oxide nanocomposites made with various functional coatings as potential magnetic carriers for biomedical applications concluded that clusters of magnetic nanoparticles could be superior to single nanoparticles in some applications [8,9,10].

Applications of magnetic nanoparticles in biomedicine are affected in different ways by the creation of linear aggregates. The magnetophoretic mobility of the system will be considerably altered as aggregates form. When paired with the size and acicular shape of the aggregates, larger particles’ increased magnetophoretic mobility may drastically modify their behaviour for use in magnetically targeted medication delivery. The toxicity of magnetic particles considerably rises after applying a magnetic field, according to recent studies by Bae et al. [11]. Their research revealed that the more significant cytotoxicity was probably caused by the increased cellular absorption caused by the creation of field-induced aggregates. Therefore, for applications with static magnetic fields, attention should be paid to building systems that limit field-induced aggregations.

Agglomerates are created when a single physical entanglement occurs between the aggregated particles and van der Waals forces, resulting in loose bonding. Because the fundamental particles that make up an aggregate are firmly bound or fused, it is stated in the ISO standards that the aggregate’s external specific surface area is less than the aggregate’s total surface area. It is also stated that because agglomerate constituents are weakly or loosely linked, the shallow specific surface area of agglomerates is equivalent to the entire surface area of those constituents.

It is important to note that IONPs in aqueous suspensions can undergo a phase transition caused by the magnetic field even at low magnetic flux density values. To do this, they can either assemble into elongated aggregates (with micrometric dimensions along the direction of the applied magnetic field) [12] or form fractal groups under the combined action of magnetic dipolar and colloidal interactions [13].

Individual nanoparticles or clusters of nanoparticles can combine to create irreversibly bound aggregates through close contact or reversibly bound agglomerates [14,15] that can be dispersed with sonication or other mixing techniques [16]. The relative strengths of the attractive and repulsive nanoparticle interactions determine whether particles agglomerate permanently or reversibly [14].

The delicate subject of flow recirculation from stents on drug release has been the subject of numerous investigations. According to Zunino et al.’s research [17], persistent recirculation emerges downstream of struts oriented transversally to the flow under steady flow circumstances. This concludes that these areas only contribute to drug accumulation in the artery despite the lengthened drug residence period. The endothelium of the blood vessel, which is made up of a thin, continuous layer of cell lining, is where nanoparticles will first come into contact after intravascular delivery, which is vital to note. As the primary focus in treating disease, including inflammatory cardiovascular disorders [18,19], the interaction of multifunctional nanoparticles with the endothelium is crucial. By allowing specific solutes and molecules to pass through, the vascular endothelium plays a selective role in the transfer of drug carriers. According to Qiu et al. [20], the vascular endothelium was more permeable when using an external magnetic field.

### Problem Description

**Figure 1:**
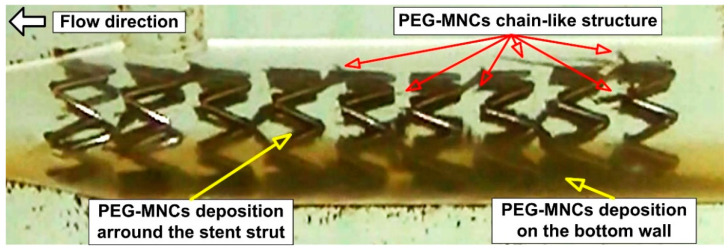
Chain-like structure development during the stent targeting processes. Magnetically induced aggregation of the PEG_MNCs in the targeted region (red arrows) at the end of the injection period of 30 s. Magnetic cluster depositions on the bottom wall of the artery model and stent struts’ coverage with magnetic clusters (yellow arrows) at the end of the injection period of 30 s. The chain-like magnetic particle structure was generated in a different part of the stent geometry in the presence of the external magnetic field. Permanent magnet positions correspond to the distance of 15 mm from the artery model bottom wall. The used magnet: rectangular NdFeB50 permanent magnet, with dimensions of 30 mm × 20 mm × 20 mm (length × width × thickness). White arrow—flow direction.

In our previous work [21], the magnetic nanoclusters produced chain-like structures in a stented vascular model during the stent targeting process (Figure 1). These magnetically built structures around the stent geometry have a significant length at the end of the targeting, with an average chain size of 0.56 mm. Removing the magnetic field at the end of the targeting procedure did not affect the spontaneous disintegration of the produced filaments into a small structure or cluster the same size as those in the injected suspension.

Without a magnetic field, the fluid flow creates a hydrodynamic force that causes these structures to disintegrate into many smaller fragments. The fluid stream picks up the resultant pieces; some are deposited distally in the stent or transmitted distally in the test circuit. From a medical standpoint, these washed particles can clog the distal capillary network and serve as a potential site for thrombus formation, which would be lethal to the patient.

The observation of agglomeration formation throughout the targeting process prompted the need to understand the cause of the formation of these structures. Thus, in study [22], based on the results of multiple experiments in the same conditions as in work [21], we determined that the agglomeration phenomenon observed during targeting is not random but rather the result of an external magnetic field. We also discovered that the stent design, particularly the presence of an external magnetic field, influences agglomeration formation. 

Optical microscopy was used to comprehend the chain-like structure generations. Also, we investigated colloidal interaction among the MNC clusters.

Our prior work established that long-range magnetic dipolar interactions emerge in an external field and prevail over colloidal ones (van der Waals, steric, and electrostatic forces). However, several questions remain unanswered, including how different magnets or field intensity influence chain generation, how the magnetorheological properties of the suspension influence structure generation, how the polydispersity index can be correlated with the behaviour and rheology of the suspension in the presence of a magnetic field, and how sonication of the samples and the density of the working fluid influence the behaviour of the suspension.

To answer the questions mentioned above, we conducted a series of new studies in addition to those undertaken in prior publications [21,22], as follows:

1. We used a new type of permanent magnet. This magnet has a length to cover the size of the stent strictly.
2. Detailed DLS investigations were performed to study the cluster distribution and polydispersity index to highlight connections with suspension magnetorheological behaviour and agglomerate generation.
3. We performed detailed optical microscopy investigations for two magnetic field intensities. It is essential that we perform the optical microscopy investigation for the same period used in the targeting processes to understand the chain development observed in the in vitro study.

## 2. Materials and Methods

### 2.1. Magnetoresponsive Nanocluster Synthesis

Our earlier works [21,22] present the PEG-coated magnetic nanoclusters. Briefly, for the preparation of PEG-MNCs, the oil-in-water mini-emulsion method was used, which involves, in the first step, the mechanical mixing of two different, non-miscible phases, an aqueous phase containing the stabilizing surfactant, PEG (PEG-2000) (1.795 g, 2% wt%), and an organic, oily phase containing the hydrophobic magnetic nanoparticles (0.5 wt% Fe_3_O_4_) (ferrofluid—magnetic nanoparticles dispersed in toluene). By using an ultrasonic finger (U.P. 400S), the two phases of emulsification take place (50% amplitude, 2 min), and very fine droplets of organic solvent (toluene) containing the magnetic nanoparticles are formed in the aqueous medium. The stabilizing surfactant (PEG) in the aqueous medium allows the formation of micelles of specific sizes in which the surfactant molecules arrange themselves with the polar end to the aqueous medium and the non-polar end to the organic phase. The formed stable mini emulsion is transferred to a larger beaker and heated (100 °C) in an oil bath under magnetic solid stirring (500 rpm) for 30 min to release the organic solvent, toluene, inside the micelles using evaporation. The as-formed magnetic clusters are magnetically separated from the reaction medium using a strong magnet, washed successively three times with a water–methanol mixture (100 mL/wash) to remove traces of reactants, and then redispersed in distilled water at known concentrations. Following that, the PEG-coated MNCs were dispersed in an aqueous carrier at preset weights to produce the investigated suspensions.

### 2.2. Characterization

#### 2.2.1. TEM Investigation

The morphology of the magnetic clusters was examined using scanning electron microscopy (STEM). For this, a microscope equipped with a cold-field emission gun, the Hitachi HD2700 (Hitachi High-Tech Corporation, Tokyo, Japan), was used. ImageJ (https://imagej.nih.gov/ij/) (accessed on 20 July 2023) was used to obtain the PEG_MNC diameter distribution. A total of 200 PEG_MNCs from three TEM images were evaluated to establish the average size distribution.

#### 2.2.2. X-ray Photoelectron Spectroscopy (XPS)

By using an XPS spectrometer with the dual-anode X-ray source Al/Mg, a PHOIBOS 150 2D CCD hemispherical energy analyser, and a multi-channel Tron detector with a vacuum maintained at 1 × 10^−9^ torr, researchers were able to determine the chemical composition (atomic concentrations) of the surface as well as the chemical state of the atoms of the coated nanoclusters PEG-MNC. XPS research used the AlKa X-ray source (1486.6 eV) running at 200 W. To enable the XPS measurements, the dispersion of particles was dried on an indium foil. The XPS survey spectra were captured at 0.5 eV/step and 30 eV pass energy. Ten scans were accumulated at a pass energy of 30 eV and a step energy of 0.1 eV to create the high-resolution spectra for the individual elements (Fe, C, and O). The Gaussian–Lorentzian product function and a non-linear Shirley background subtraction were used in CasaXPS v10 software for data processing and curve fitting.

#### 2.2.3. Dynamic Light Scattering (DLS) Measurements

A Malvern Zetasizer Nano-ZS device (Malvern Panalytical Ltd., Malvern, UK) outfitted with a He-Ne laser (λ = 633 nm, max 5 mW) and operating at a scattering angle of 173° was used to quantify the particles’ hydrodynamic size and zeta potential. Before the measurements, the samples were diluted to a 0.1 mg/mL concentration. One millilitre of particle suspension was used in each measure.

#### 2.2.4. Magnetic Characterization

The magnetization curves of the PEG-coated clusters were measured using a vibrating sample magnetometer (VSM 880-ADE Technologies, Westwood, MA, USA) at room temperature in the field range of 0–1000 kA/m.

#### 2.2.5. Rheology

The magneto-viscous characteristics of the pegylated nanoparticles were tested at 25 °C in both the presence and absence of a magnetic field using a rotating rheometer (Anton Paar MCR 300 Physica, Anton Paar GmbH, Graz, Austria) with a 20 mm diameter plate–plate magnetorheological cell (MRD 170/1T-SN80730989). In this cell, a perpendicular magnetic field is applied to the sample layer situated between the plates. A Hall probe installed under the bottom plate of the MR cell measures the magnetic flux density of the applied magnetic field.

### 2.3. Magnetic Field Generation

MNC retention can be measured in vitro to estimate what would happen in vivo. Thus, it is possible to investigate the effects of several factors on the efficiency of magnetic targeting [23], including magnet configuration, flow velocity, particle surface features, separation from the magnetic pole, and particle size.

The concept of the present study, which is based on our prior findings [24,25], is to use a single external permanent magnet system to create a strong magnetic field that will be used to magnetize, direct, and deliver the MNCs in the stented vascular segment.

**Figure 2:**
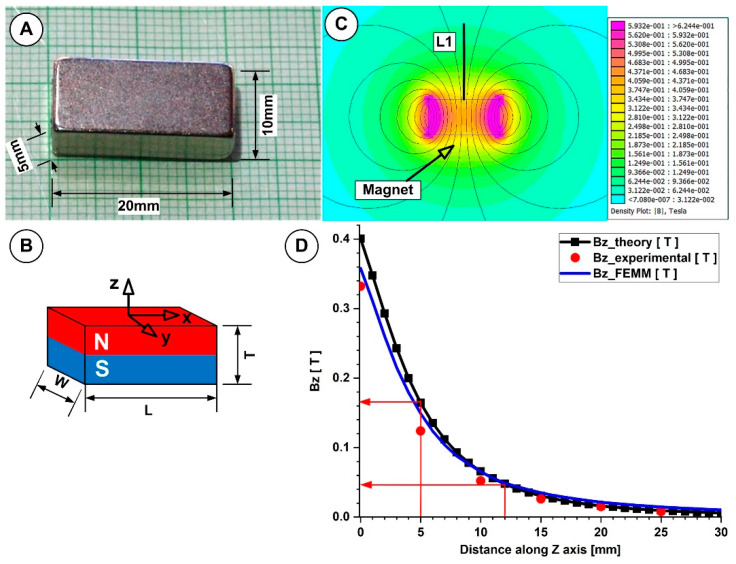
A magnetic field generated with the NbFeB52 permanent magnet was used in the experimental investigation. (**A**) The dimension of the used magnet and axis association. (**B**) A used permanent magnet has polarization along the Z-axis. (**C**) Numerical investigations of the magnetic field generated with the NdFeB52 magnet. (**D**) *B_z_* evolution function of the magnet surface distance. Comparison between theoretical, experimental, and numerical results.

The rectangular NdFeB52 permanent magnet (commercial notation N52: Neodymium 52), which has dimensions of 20 × 10 × 5 mm (length × width × thickness) and a maximum energy product (BxH) of 52 MGOe, was utilized to create the magnetic field in our experiment (Figure 2).

#### B-Field Measurement

Using a Tesla meter (Model 5080, F.W. Bell Gaussmeter, Milwaukie, OR, USA) placed using a micrometre, the actual B-field strength was measured along the magnet’s central axis at various points. The measurement error of the micrometre was calculated to be 0.15 mm, while the measurement error of the B-field was taken from the Tesla meter manual. With a peak magnitude of 405 mT and a drop-off to 0.08 mT at 3 cm from the magnet’s base, the B-field magnitudes along the central axis of the interest fell exponentially with distance (Figure 2D). The magnetic field’s most significant spatial gradient was close to the magnet’s edges (Figure 2D). In the present experiment, to investigate the influence of the magnetic field on MNC targeting and the agglomeration phenomena, the magnet position ranged between 5 and 12 mm from the vessel wall (corresponding to magnetic field intensity of 0.42 and 0.183 T, respectively; Figure 2D, red arrows). 

We compare the B-field distribution obtained from analytical solutions, numerical simulations, and experimental investigations to obtain a clearer picture of the generated magnetic field.

With [26], Equation (1) was used to obtain the analytical solution for the B-field distribution along the magnet’s central axis at different distances (Line L1 in Figure 2C).

(1) $$B\left(z\right)=\frac{B_{r}}{\pi }\left(\tan^{-1}\frac{WL}{2z\sqrt{4z^{2}+W^{2}+L^{2}}}{-\tan}^{-1}\frac{WL}{2\left(z+T\right)\sqrt{4{\left(z+T\right)}^{2}+W^{2}+L^{2}}}\right),$$

where *W* is the magnet width, *L* is the magnet length, *T* is the magnet thickness, *B_r_* is the magnet residual flux density, and *z* is the distance from the magnet surface (where *z* ≥ 0) on the magnet’s centreline.

The examined permanent magnet was numerically simulated using the freeware Finite Element Method Magnetism (FEMM) version 4.2 (accessed on 10 June 2023, at http://www.femm.info/wiki/HomePage).

The experimentally obtained B-field values and the corresponding analytically and numerically calculated values were in good agreement, as shown in Figure 2D.

## 3. Results

### 3.1. PEG-MNC Size and Morphology

**Figure 3:**
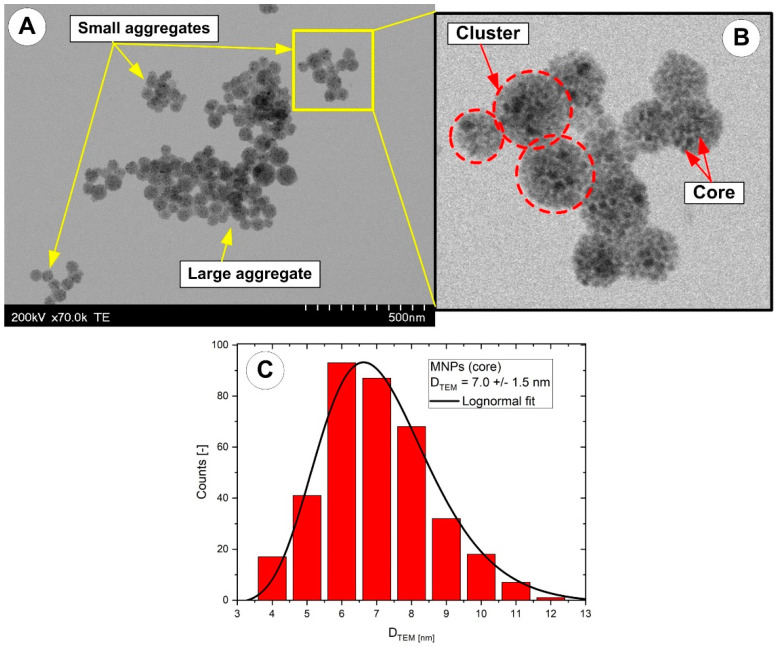
(**A**) Representative TEM image of the PEG-MNC clusters and aggregates. The aggregates show chain-like and close-packed morphologies (yellow arrows). (**B**) Detail regarding the spherical clusters (red circle) and core inside the cluster. TEM size histograms for core nanoparticles (**C**).

The nanoclusters possess a well-defined spherical shape with a core–shell structure consisting of a cluster core with closely packed magnetite nanoparticles (Figure 3A,B). The PEG_MNCs’ size was measured with direct counting from TEM micrographs and showed diameters ranging from 40 to 120 nm. In turn, the morphologies of the small nano-chain aggregates (Figure 3A, bottom chain) magnetically assembled from MNCs and chemically fixated show predominantly single-particle chains of a few nanoclusters. Counting particle lengths from TEM micrographs yields a distribution of nano-chains in the range of 4–8 nanoclusters with a mean of 6 ± 3 nanoclusters.

Also, the micrographs indicate that the visualized structures had arbitrary morphologies ranging from chain-like to closely packed, creating both small and large aggregates (Figure 3A). Furthermore, TEM micrographs showed that the dimensionality and size of these structures varied greatly and could contain many MNCs.

ImageJ was used to obtain the PEG_MNC and the core (magnetite nanoparticles) diameter distribution (imagej.nih.gov/ij/; accessed on 15 June 2023). Each sample’s histogram, created by measuring 200 clusters over three TEM images, was fitted to a lognormal distribution function.

Figure 3C displays the diameter distribution of the core magnetic nanoparticles. With an average core size of 7 ± 1.5 nm and an average cluster size of 62 ± 17 nm (presented in our previous work [22]), TEM observations support the multicore flower-like shape of synthesized particles.

It is vital to note that the sample drying process greatly influences TEM micrographs. Dewetting reduces the contact area between clusters, and attractive interparticle forces cause aggregation, aided by drying [27].

During the TEM sample preparation, the polymer chains’ conformation may alter because of the increasing pressure, which could cause the coating layer to contract.

In the present work, we use the term “aggregate” for this structure because the investigated structure results from the formation of compact masses of irreversibly linked clusters, per the definition presented in [13].

It is critical to distinguish between ‘real’ aggregates in the sample and ‘developed’ aggregates developed during sample processing. For this, DLS was used to evaluate the materials in their suspended natural condition, and the results were compared to those of TEM. The results are presented in the following sections.

### 3.2. The Surface Chemical Analysis of the Magnetic Cluster

The successful coating of the magnetic clusters with PEG was shown with the XPS analysis (detailed investigations were presented in [22]). The C 1s, O 1s, and Fe 2p core-level XPS high-resolution spectra for PEG_CMC were investigated. The contributions from the peaks corresponding to specific groups of the surfactant, oleic acid, and the PEG coating of the clusters are seen in the deconvolution of the C 1s and O 1s spectra. Three components, corresponding to C-C, C-H (284.7 eV), C-O (286.6 eV), and O-C=O (288.9 eV), were found to suit the C 1s spectrum best. Three elements that are attributed to Fe-O (530.2 eV), C-O (531.8 eV), and O-C=O (533 eV) may be seen in the O 1s spectrum. The doublets Fe 2p3/2 and Fe 2p1/2 can be found in the Fe 2p spectrum.

### 3.3. DLS Characterization of the PEG-MNCs’ Suspension

The PEG-MNC’s stability was assessed using DLS measurements. The Malvern Zeta Sizer evaluated the studied samples for zeta size and potential. Three crucial properties of the final PEGylated MNC are revealed with dynamic light scattering (DLS): size distribution, zeta potential (ZP), and hydrodynamic diameter (zeta-average of the MNC).

ZP levels typically fall within the −100 to +100 mV range [28]. Colloidal stability can be predicted based on ZP magnitude. A high level of stability is naturally present in the ZP of NPs with values of −25 or + 25 mV. High ZP denotes powerfully charged particles, which inhibit particle aggregation by preventing electric repulsion. Attraction triumphs over repulsion when the ZP is low, and the mixture is likely to agglomerate, coagulate, or flocculate because of van der Waals interparticle attraction [28].

**Figure 4:**
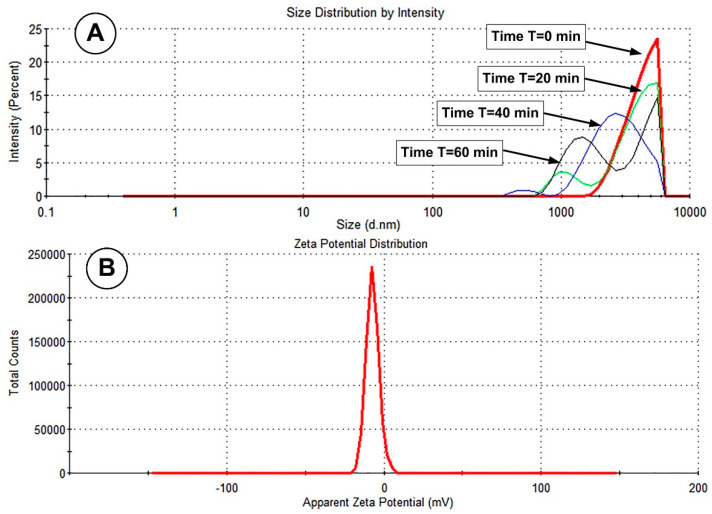
(**A**) Size distribution by intensity for the PEG-MNC aqueous dispersion corresponding to different time steps after the vigorous stirring of the suspension. (**B**) Zeta potential corresponding to the investigated PEG-MNCs’ suspension.

The MNCs used in our studies were coated with a PEG2000 layer, giving them unique surface charges and chemical characteristics. In deionized water, the PEG-MNC assumed a dispersed colloidal state with an average hydrodynamic diameter of 4527 nm (Figure 4A time T = 0 min) and a negative surface charge of −7.22 mV (Table 1, Figure 4B). The sample’s aggregation was measured using the polydispersity index (PDI), which was discovered to be 0.229.

The hydrodynamic size is substantially more significant when compared to the matching TEM observations, indicating the existence of aggregates in this solution. Furthermore, the polydispersity index (PDI), calculated as the ratio of the standard deviation square to the mean size, was discovered to be 0.229. Due to the existence of bigger aggregates, the PDI of the PEG_MNC solution is relatively high.

In Figure 4A, it is evident that there are large agglomerates (micro-size structures) in the PEG-MNC aqueous dispersion, which exhibits a monomodal distribution and polydispersity. According to descriptions in the literature, a polydispersity index (PDI) value of less than 0.1 may indicate a high degree of homogeneity in the particle population, whereas high PDI values indicate a wide size range or possibly multiple populations [29].

It is crucial to give precise feedback regarding the time frame involved in this process to employ the DLS to monitor the aggregation kinetics of MNCs [30]. In our investigations, the DLS measurements track the PEG-coated MNCs’ colloidal stability in an aqueous dispersion over 60 min (Figure 4A).

As seen in Figure 4A, during the first DLS measurement, which was performed precisely after vigorous stirring, vast clusters of agglomeration occurred with an average hydrodynamic diameter of about 4572 nm. Stronger magnetic attraction exists within the groups because the magnetic attraction between particles grows with a particle radius to the sixth power, further causing flocculation. The intensity-weighted size distribution shifts to bimodal after 20 min, as seen in Figure 4A. The size of the individual agglomerates (first peak) that remain suspended decreases over time (4572 nm at the time T = 0 min, ≈3400 nm at the time T = 20 min compared to ≈2600 nm at the time T = 60 min), indicating that the initial clusters take some time to form large agglomerates with a bimodal distribution (such as that at time T = 60 min).

#### 3.3.1. PEG-MNCs’ Sedimentation Kinetics

As shown in the next paragraph, sedimentation kinetics were studied using DLS measurements of the hydrodynamic diameter in two stages.

**Figure 5:**
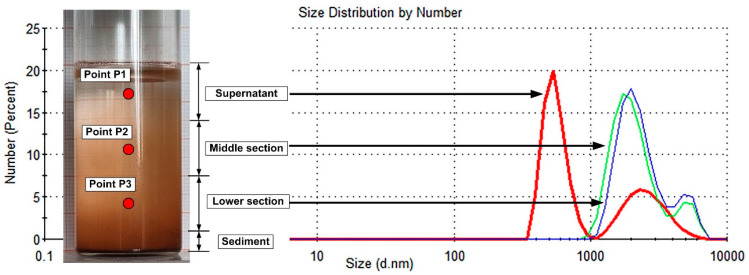
Particle size distribution by number obtained using DLS measurements at 60 min after manual stirring. Measurements were performed at three points corresponding to different stages of cluster sedimentation. Points P1, P2, and P3 represent the position where the samples were taken and used colloid: PEG_MNCs dispersed in distilled water.

Stage one: The settling behaviour of the suspensions of PEG-MNCs in the distilled water was checked using DLS measurements at 60 min after manual stirring (Figure 5), with measurements of the hydrodynamic diameter distributions of the suspended PEG_MNC population in the different segments of the investigated tube (the test tubes contain 20 mL of a 5% mass concentration PEG-MNC aqueous suspension).

Figure 5 shows that the peak position moved to be smaller for the supernatant region, demonstrating that the sedimentation processes are progressing. Small and large aggregates and agglomerates are present in the supernatant, but the number of the small structures is much larger than the large ones. The particle size distribution in the middle section and the lower area is very similar but indicates the presence of the gravitational sedimentation processes with the bimodal size distribution. More important is that, at the end of 60 min, a consistent layer of PEG-MNCs was deposited on the bottom of the tube.

Phase two: DLS measurements checked the settling behaviour of PEG-MNC suspensions every 10 min for 60 min after vigorous manual stirring. The measurement was performed for samples from the position corresponding to Point 2 in Figure 5.

**Figure 6:**
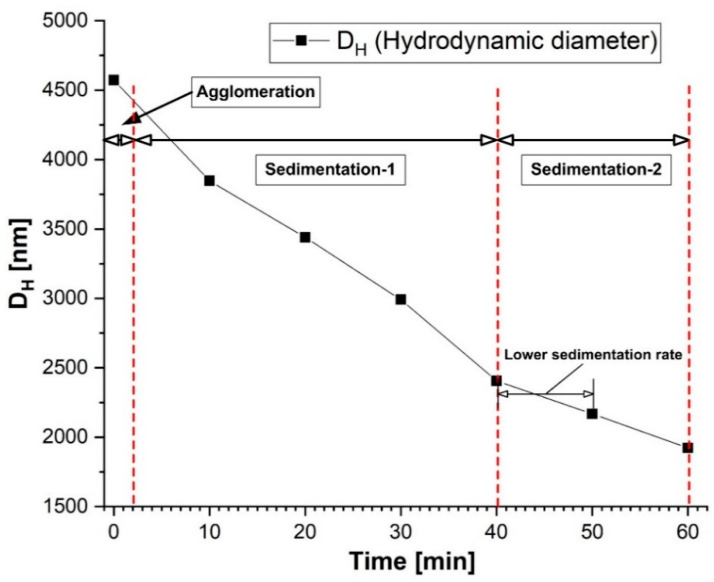
Sedimentation curve for the 5% mass concentration PEG-MNCs dispersed in distilled water.

The PEG-MNC suspensions’ sedimentation kinetic curve is shown in Figure 6. In Figure 6, the sedimentation profile is divided into three areas: agglomeration, sedimentation-1, and sedimentation-2, according to the findings presented in [31]. When the aggregates or agglomerates reach a threshold size, they rapidly deposit (“sedimentation-1” segment in Figure 6). After a while, the sedimentation rate can decline, consistent with the sedimentation of various structures that fail to reach the required critical size [31].

After the investigation period, the DLS measurement verified the presence of a structure with an approximate hydrodynamic diameter of 1800 nm (Figure 6), showing that a significant portion of the PEG-MNC agglomeration or aggregate population had settled on the cuvette’s bottom.

#### 3.3.2. Effects of Sonication on Aggregate Size

As was previously mentioned, a stable colloid may be mostly made up of individual clusters, aggregates, and agglomerates; all these structures tend to clump together and settle over time. The sonication process was used in this work as a method to enhance the dispersion of nanoparticles in liquids. It is well recognized that a properly timed treatment can aid in dispersion and uniformity of the suspension, but extended sonication times can also cause ultrasound-driven aggregation [32].

**Figure 7:**
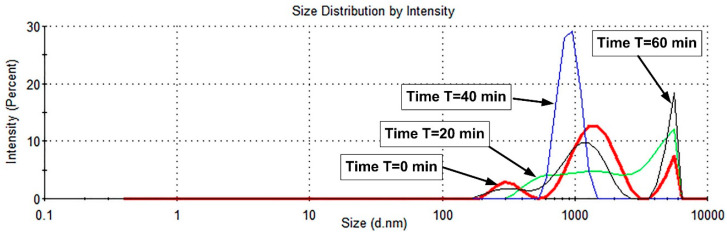
Particle size distribution by intensity of the 5% mass concentration PEG-MNCs dispersed in the distilled water at different time intervals after 15 min of bath sonication.

A bath-type sonicator (Bandelin RK 100 H ultrasonic bath, Bandelin electronic GmbH & KG, Berlin, Germany, 320 W) operating at room temperature distributed the PEG-MNCs in the base fluid. The sample received 2880 J of energy during the 15 min sonication process. DLS measurement is used to track the colloidal stability of the PEG-coated MNCs in the aqueous dispersion after 15 min of bath sonication for over 60 min (Figure 7). 

As shown in Figure 7, the initial suspension (time T = 0 min, performed exactly after sonication) exhibited a broad polydisperse and multimodal size distribution primarily in the 530 nm–3 μm range, with two minor fractions of particles, the first of which had a peak centre near 230 nm (range of 190 to 530 nm) and the second of which had a peak centre near 5.5 μm (range of 3.5 to 6.4 μm). As can be seen in Figure 7, the initial PEG-MNCs’ profile changed throughout the investigation from a multimodal distribution (times T = 0 min and T = 60 min) to a bimodal distribution (T = 20 min) and a monomodal distribution (T = 40 min), displaying both a microscale agglomerate and a nanoscale fraction.

Contrary to the literature [29,32], bath sonication in our situation does not result in uniformly sized particle dispersions or a considerable reduction in the PEG-MNC aggregate size.

### 3.4. Magnetic Properties of the Magnetoresponsive Nanocluster

One of the most crucial conditions for successful applications in biomedicine, including magnetic targeting, is the strong magnetic moment of the functionalized multicore carriers [33].

Magnetic experiments with a vibrating sample magnetometer (VSM) reveal that the synthesized nanostructures’ colloidal and powder forms behave superparamagnetically, as demonstrated in our prior work [22]. Also, measurements show no hysteresis, and it suggests that the magnetic moments of the nanoparticles packed into the centres of nanoclusters and nano-chains are unblocked using heat.

The saturation magnetization for the PEG-coated MNCs is 55 emu/g, and for the water-based suspension, it is 142 memu/g. These are both less than the values for bulk magnetite, 92 emu/g. These are the consequences of the PEG layer, water, and the induced non-magnetic layer (due to the surface effects of the nanoparticles embedded in the cluster) [34].

### 3.5. PEG-MNC Aqueous Dispersion Rheological Properties

The start and course of many cardiovascular diseases are influenced by the mechanical blood vessel wall behaviour and blood flow properties [35]. While operating more like a Newtonian fluid in the large arteries, blood behaves differently in the small/capillary arteries [36]. The wall shear stress (WSS) results from friction between the flowing blood and the endothelial surface of the artery wall.

In the current study, the possibility of particle targeting for a stented artery was explored for the WSS value in a range of 0.1 s^−1^ to 1000 s^−1^, corresponding to the blood flow in the stented artery [37].

#### 3.5.1. Rheological Properties of the PEG_MNCs’ Aqueous Dispersions

Parallel plate geometry was used to apply steady shear strain to the suspensions. A shear rate ramp up in the 0.1–1000 s^−1^ range was used to obtain the viscosity curves of apparent viscosity (η) vs. shear rate ($\dot{\gamma }$). The magnetorheological accessory produced a perpendicular external magnetic field to the shear flow. The temperature for all rheological testing was 25 °C. The variations in the shear viscosity of PEG_MNCs’ aqueous suspension as a function of the shear rate without the magnetic field and for two distinct magnetic field intensities, 42 and 183 mT, were presented in our previous work [22]. 

##### Viscosity Changes in the Absence of the Magnetic Field

The PEG_MNC aqueous suspension shows the shear thinning typical of multicore particle suspensions [38,39], brought on with the clusters’ aggregation or agglomeration. The van der Waals attraction force between the clusters is what propels the agglomeration in the absence of a magnetic field. As can be seen, as the field intensity increases, the magnetic force takes precedence over the hydrodynamic force, increasing viscosity as the field-induced aggregates get more robust and resist shear. The characteristics of the viscosity curves indicate areas of varying slopes that represent the distinct structural evolution in the sample. Also, these characteristics exhibit a non-Newtonian nature with a progressively emerging typical shear thinning behaviour. Because the investigated suspensions were non-Newtonian [39,40], we used the Carreau model [41] (Equation (2)) to fit the measured viscosity curves.

(2) $$\eta (\dot{\gamma })=\eta_{\infty }+(\eta_{o}-\eta_{\infty }){\left[1+(C\dot{\gamma }{)}^{2}\right]}^{-p}$$

where *C* (s) is the Carreau constant (the slope of the viscosity curve on the log–log scale at high values of the shear rate), *p* (−) is the Carreau exponent, and *η*_0_ and *η*_∞_ (Pas) are the viscosities at infinitely low and infinitely high shear rates, respectively.

As presented in our previous work [22], the increased viscosity of the PEG_MNC suspensions at low shear (0.1–10 s^−1^) is caused by this network of agglomerated particles. Shear forces break up the particle network once the suspension starts to flow and the shear rate rises. As a result, as the shear rate increases, the agglomerates’ size continuously decreases, which causes the viscosity to decrease. When the viscosity reaches a constant value, only shear forces can shrink the size of the clusters.

##### Magnetorheological Investigations

Both the magnetorheological effect (MRE), which increases shear stress when an external magnetic field is applied, and the magneto-viscous effect (MVE), which increases viscosity when an external magnetic field is used, were studied in the presence of the magnetic field. These effects are critical to developing a strategy for utilizing these intelligent fluids.

This network of agglomerated clusters contributes to the greater viscosity (*η* ≈ 1–10 Pas) of the MNC suspensions at low shear. On the other hand, viscosity in the low shear rate area rapidly dropped, which indicates that the structures of the dispersed PEG_MNCs formed under the external magnetic field persisted until the shear rate reached the value of 1000/s.

**Figure 8:**
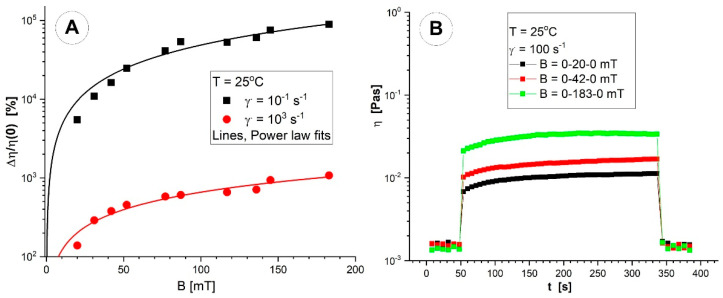
Rheological properties of the PEG_MNCs’ aqueous suspension at the temperature of 25 °C. (**A**) Magneto-viscous effects’ function of different magnetic flux densities for two different shear rates (0.1 and 1000 s^−1^); (**B**) viscosity curve function of time for different magnetic flux densities.

The MVE on the range of low shear rates (0.01 s^−1^ to 10 s^−1^) is found to be almost independent of the shear rate, but it significantly decreases at high speeds as cluster agglomerations are destroyed. Figure 8A shows the MVE’s dependence on the strength of the magnetic field for various shear rate values. The MNC agglomerates are destroyed for higher shear rates, and the MVE decreases. The observed MVE behaviour is a consequence of the multicore nature of the magnetic component, resulting in a high induced magnetic moment of particles, favouring their structuring in a magnetic field corresponding to the findings presented in [42].

Figure 8B illustrates the test results aiming at the viscosity behaviour as a function of time or the quickness of the development or destruction of cluster agglomerations while applying or halting the external magnetic field. The test contains three intervals at which a low shear rate is kept constant—so as not to disturb the formation of particle agglomerations too much. The torque has values high enough (>5 μNm) for the reproducibility of experimental data. On the first and the last interval, the magnetic field intensity was fixed at the value of 0 (with six experimental points each); on the second interval, the values of field intensity B were 20, 42, and 183 mT and were fixed in turn. The temperature was set as T = 25 °C. The magnetic field intensity was chosen following the results presented in Figure 2.

To better understand how quickly cluster agglomeration occurs when an external magnetic field is applied, the measurement time in this test was set at 6 s/point for intervals with no magnetic field and 8 s/point for intervals with one. Due to low sample viscosity values, shorter measurement periods or points could not be specified. The structures are seen to form within 6 s. In the absence of a magnetic field, slow agglomeration persists throughout the application of a magnetic field, and for a field intensity of 183 mT, the agglomeration phenomenon reaches saturation 150 s after the magnetic field application. The formation or destruction of particle agglomerations happens very quickly. For all investigated magnetic field intensities, the suspension viscosity practically returns to its value before applying it after the magnetic field is turned off.

#### 3.5.2. Effects of the Carrier Fluid Density

From a macro-rheological perspective, it is known that the haematocrit (concentration of red blood cells) and blood viscosity are directly correlated [43], which means that an increase or decrease in the RBC concentration affects blood viscosity values as well as its non-Newtonian behaviour [44]. At a shear rate of 0.1 s^−1^, the viscosity of the same blood can be 60 cP (0.06 Pas), whereas, at a shear rate of 200 s^−1^, it would be 5 or 6 cP (0.005 or 0.006 Pas) [45]. This indicates that blood viscosity varies in the microcirculation, major arteries, and veins, where shear rates can range from a few to more than 1000 s^−1^ [46]. At low shear rates (such as veins) as opposed to high shear rates (such as arteries), the effect of haematocrit on blood viscosity is significantly more significant [47].

Considering the previous, we sought to determine how the variation in the carrier fluid (CF) density affected the rheological characteristics of the model suspension. We produced CF with 1055 and 1068 kg/m^3^ densities to conduct this, respectively. The density was changed by adjusting the glycerine–water ratio in the CF composition. Our study’s carrier fluid (CF) consisted of glycerol–water solutions with a density (1055 kg/m^3^) equivalent to blood. The rheological characterization of CF was detailed in our previous work [20,21]. 

**Figure 9:**
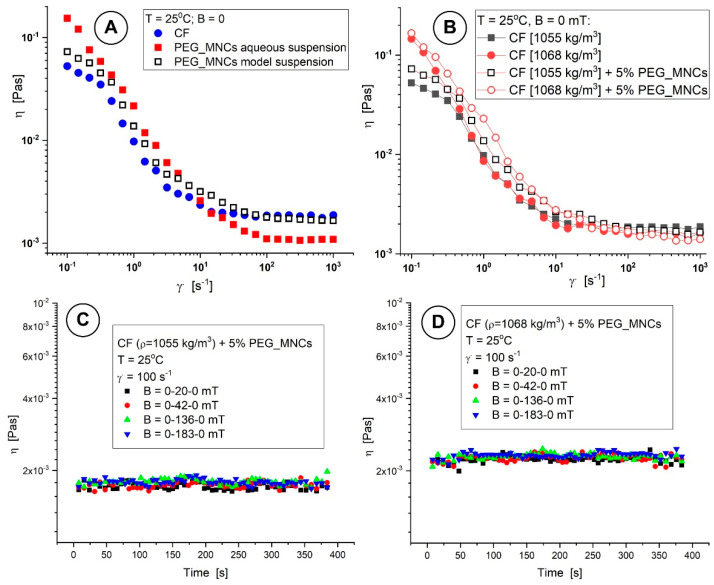
(**A**) Comparison between CF, aqueous, and model suspension viscosity curves at T = 25 °C. (**B**) CF and model suspension viscosity curves at T = 25 °C for different density values. (**C**,**D**) Viscosity curve function of time for different densities of the carrier fluid (CF) for the model suspension (CF + 5% PEG_MNC) corresponding to different magnetic flux densities.

Figure 9A depicts the rheological properties of CF, PEG_MNC aqueous suspension (distilled water + 5% PEG_MNC), and model suspensions (CF + 5% PEG_MNC). The presence of PEG_MNC has a considerable influence on the suspension viscosity curve for the aqueous suspension in the low and high shear regions. Still, the viscosity curve for model suspensions exhibits a progressive increase over the whole inquiry range. Also, Figure 9A shows that all the examined suspensions exhibit shear thinning behaviour at relatively low shear rates (<10 s^−1^). Figure 9B demonstrates that the model suspension’s (CF + 5% PEG_MNCs) rheological behaviour is essentially unaffected by changes in the CF density. At low levels of the shear stress, a variation in behaviour between the suspension model and the CF is seen. It is significant to note that increasing the carrier fluid’s density does not impact the model suspension’s magnetic viscous behaviour (Figure 9C,D).

The glycerol–water PEG_MNC dispersions were selected because they allowed us to assess a slight change in viscosity when a magnetic field was applied and examine the magnetorheological properties. This is the most intriguing aspect of how we conducted this experiment.

We employed this blood analogue fluid (water–glycerol solutions) during the experimental examination to guarantee that the rheological features of the carrier fluid accurately recreated blood rheology. The flow field across the test portion must exhibit the same characteristics as normal blood flow (a viscous flow). Fluid flow is an essential predictor of PEG_MNC aggregation surrounding the implanted stent, as demonstrated in our early works [21,22]. Our investigations showed that these dispersions are stable for 1 h, which is long enough for targeted experiments and viscosity measurements.

Because the viscosity of a glycerol aqueous solution is highly sensitive to temperature changes, neither the temperature nor the applied magnetic field must vary during the targeting and measuring time. As a result, all experimental measurements are carried out in an air-conditioned room. Furthermore, to prevent working fluid temperature change, measurements are taken in an open circuit where the fluid is not recirculated. Also, we used DC (direct current) magnetic fields, which do not transmit heat to the test section.

### 3.6. Kinetics of the PEG_MNC Chain Formation in the Magnetic Field

Any ferrofluid will inevitably have particle-size polydispersity, which is crucial for creating structures generated with the field [48]. In a polydisperse fluid, the particle size, shape, or strength of the interaction between the components might differ [49].

The field-induced microstructures created with the magnetic field were examined using an optical microscope. 

We investigated two approaches for optical microscopy research. In the first case, we used a low-intensity magnetic field (42 mT); in the second, we used a magnetic field with a strength of 124 mT. Each field intensity corresponds to the different positions of the permanent magnet, as shown in Figure 2. This technique was designed to investigate the effect of external magnetic field intensity on aggregate and agglomerate formation. Both scenarios used an aqueous suspension containing 5% mass concentration PEG_MNC. All solutions were vigorously stirred before testing, and the investigated samples were collected from the same region of the tube (middle section, as shown in Figure 5). More importantly, these analyses were conducted in both situations for the same injection period (30 s) employed in the targeting methods to understand agglomeration development better.

**Figure 10:**
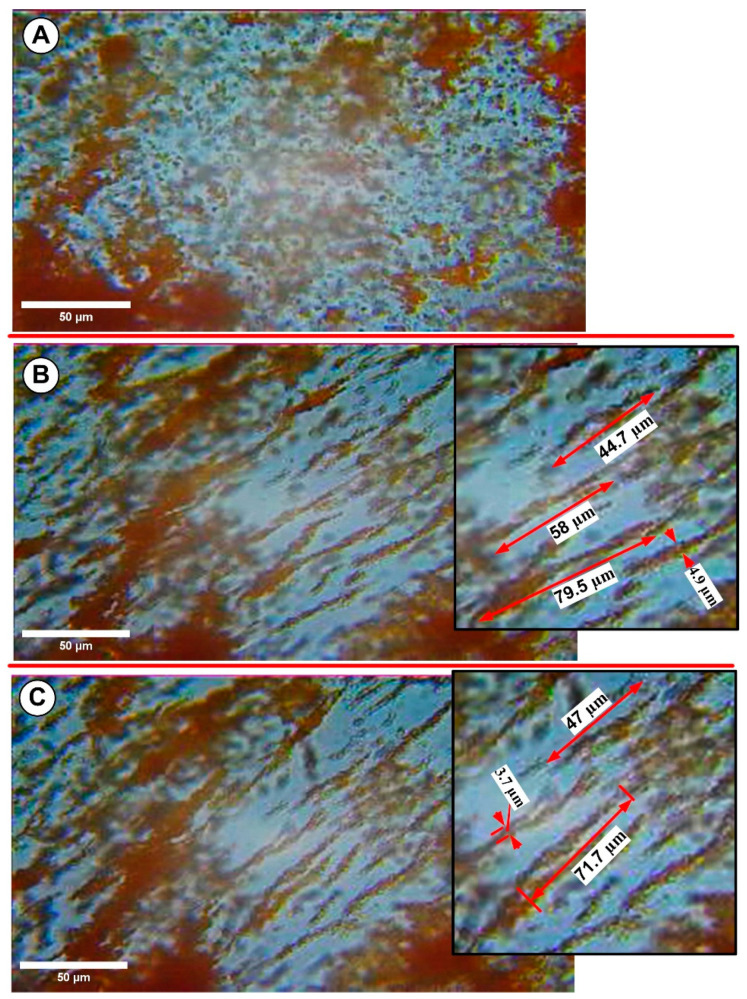
Optical microscopy investigations of the PEG-MNC aqueous suspension phase condensation phenomena induced in the presence of the external magnetic field. (**A**) Suspension without a magnetic field. (**B**) The figure shows the large PEG_MNC agglomerates generated under the action of the externally applied magnetic field of intensity H = 47 mT—detail regarding the length and thickness of these large, generated structures (chains). (**C**) The PEG_MNC agglomerates after turning off the magnetic field. Detail shows that the suspension contained large, micro-sized cluster agglomerates after turning off the magnetic field. All measurements of the agglomerate’s length were processed using ImageJ software—scale bar: 50 μm.

As shown in Figure 10A, without a magnetic field, the dispersion’s superparamagnetic nanoparticles move randomly according to Brownian motion. When exposed to an external magnetic field, the nanoparticles’ induced magnetic moments align with the magnetic field’s direction. 

Figure 10B shows the formation of magnetically induced large MNC filaments (agglomerates) that are field-oriented. The initial suspension is subjected to the magnetic field for 30 s, the same amount of time employed for stent particle targeting. During this time, the PEG-coated MNCs self-assembled into linear agglomerates in the magnetic field’s direction (see Supplemental Video). Also, the filaments do not spontaneously disintegrate once the magnetic field is removed; instead, they become randomly orientated, as presented in Figure 10C. Figure 10C shows that these large structures did not shrink or fragment once the magnetic field was removed. The Supplemental Movie provides a detailed presentation of the creation and development of these structures.

Given the size of these large structures in the range of several micrometres generated under the influence of magnetic fields, it is reasonable to assume that the observed behaviour is closely related to the magneto-viscous properties outlined in earlier sections. As a result, quantifying structure formation is critical to analyse the influence of external magnetic field intensity and duration on the size of chain-like shapes.

#### Effect of the Magnetic Field Intensity

We used optical microscopy to learn more about the interaction of PEG_MNCs in the presence of a magnetic field. This experiment aims to demonstrate the effect of the increasing magnetic field strength on agglomeration processes.

The optical microscopy experiments were carried out under the following conditions: a magnetic field intensity of 124 mT (corresponding to a magnet position of 7 mm from the artery bottom wall), a magnetic field application period of 30 s (same as in the previous experiment), and a vigorously stirred sample of the PEG_MNC aqueous suspension.

**Figure 11:**
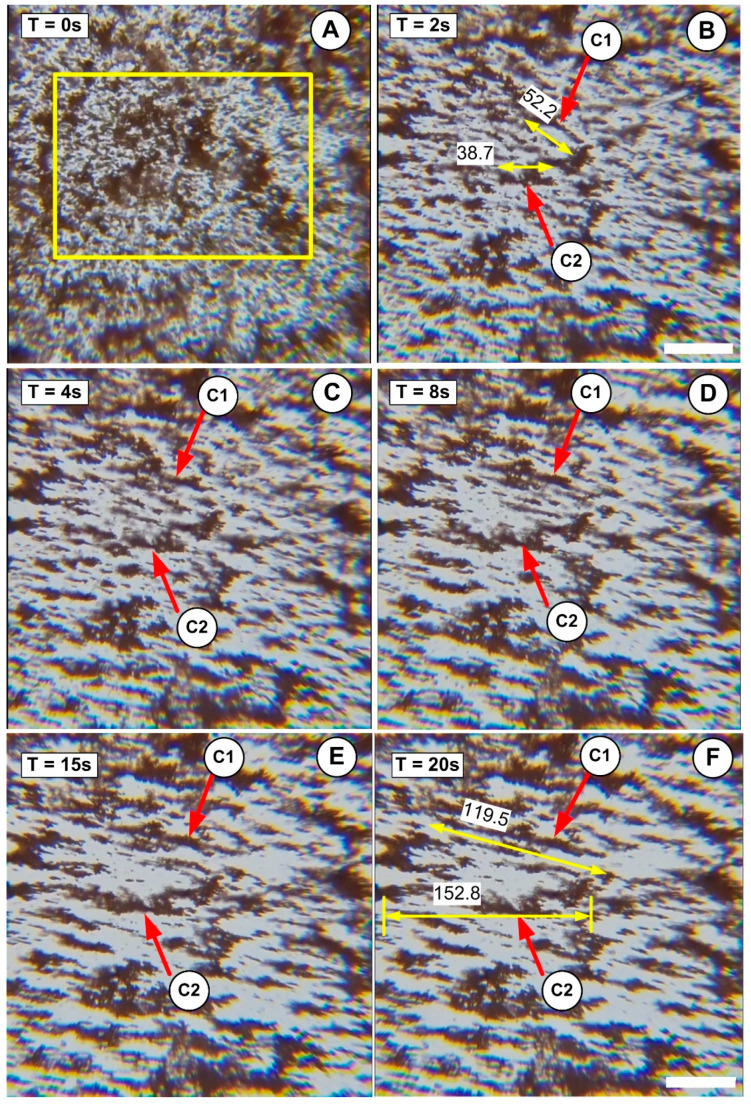
Optical microscopy images of the PEG_MNC transition from a dispersed stage to chain-like structures in the presence of the external magnetic field. (**A**) Suspension in the absence of the magnetic field. (**B**–**F**) Stages of the chain structure’s development after the magnetic field was switched on. The investigated period is 30 s, under the action of the externally applied magnetic field of intensity H = 124 mT. (**B**,**F**) detail the length and thickness evolution during the investigated period for two agglomerates (chains), C1 and C2. (**F**) shows the sizeable micron-size chain structure oriented in the field direction at the end of the investigation period—scale bar: 50 μm.

Figure 11 shows images of PEG_MNC clusters in aqueous suspension after field application. The PEG_MNC appears diffused in the carrier fluid without a magnetic field (Figure 11A). When the magnetic field is activated, the MNCs migrate and form agglomerates directed in the field direction, as seen in Figure 11B–F.

Figure 11A demonstrates that the sample initially showed no agglomerates but showed pre-existing cluster aggregates. The size distribution and volume fraction of particles and the applied field’s strength determine the kinetics of field-induced aggregation in ferrofluids. A ferrofluid system will be thermodynamically stable without a magnetic field [38]. When subjected to a magnetic field, however, the stability is lost, and to re-establish equilibrium, both clusters and aggregates begin to aggregate/agglomerate to produce two distinct phases, namely carrier fluid and the formed structures.

This separation begins with a brief nucleation step in which a few particles or pre-existing aggregates initiate particle agglomeration in the presence of an external magnetic field.

As shown in Figure 11B and more clearly in the Supplemental Video, a short but thick structure emerges a few seconds after the rapid initiation of the magnetic field. These formations (agglomerates) are oriented in the field direction and move in the carrier fluid. During the following several seconds, these primary agglomerates adhere together head to tail to create large (>150 μm) and practically stationary needle-like secondary agglomerates (Figure 11F). The agglomerate’s size grows with time (Figure 11B–F). Individual agglomerates develop relatively quickly (between 2 and 8 s), owing to the adsorption of magnetic clusters or tiny aggregates from the surrounding fluid. Because of their dipolar contacts, the surrounding aggregates consolidate and form a long-size structure (agglomerates) in the following period (between 10 and 20 s, Figure 11D,F).

The shape and size of the created agglomerates remain nearly constant between 20 and 30 s. These findings underline that the generated structures have achieved their maximum length and are in equilibrium.

## 4. Discussion

The investigated PEG_MNC suspensions possess significantly larger particles, as evidenced with DLS results. The larger particles have stronger dipolar strength [38]. This system’s initial susceptibility and magnetization curve strongly depend on the number of bigger particles. When the number of bigger particles rises, the magnetization of the system grows quicker, even in weak fields, resulting in a higher initial susceptibility [38,48]. Larger particles have been shown to play a significant function as condensation centres in the production of nuclei [12,50]. When the chain length exceeds a certain threshold, it attracts the next tiny cluster (including both big and small particles). It creates a thicker column, which might be considered the nucleation location (according to [12]). As a result, even at modest field strengths, aggregates act as nucleation sites, initiating chain development. As field strength increases, the chains lengthen, increasing the aspect ratio. Chain zippering produces long and dense columnar formations. 

However, in a system with high polydispersity, the larger particles can operate as nucleation centres even at very low field strengths, implying that aggregation kinetics are much faster with significantly lower activation energy, resulting in a higher nucleation rate.

In conclusion, the created agglomerates were developed around the existing large structure. These initial large structures functioned as condensation sites for heterogeneous nucleation of free clusters and existing aggregates.

Given that our ferrofluids contain oleic-acid-capped magnetic nanoparticles (as described in [21]), we hypothesized that the occurrence of cluster aggregations (as shown with the TEM observations, Figure 3A) is most likely owing to a non-uniform surfactant coating on their surface. Our idea is consistent with the findings provided in [51]. Furthermore, because the particles are functionalized, they cannot come into direct contact with one another, even in an external magnetic field. As a result, solvent molecules will become trapped between the nanoparticles, forming an aggregation. More solvent molecules are trapped between aggregates when the chain density is large [51]. This idea is consistent with that, as stated before in [22], MNCs are soldered in aggregates due to strong contacts between polyethylene glycol (PEG) shells or the collective encapsulation of many MNCs within PEG. 

Rheological studies of the PEG_MNC suspension at low field strength (42 mT, Figure 8A) revealed a considerable increase in viscosity across the whole examined shear domain. Furthermore, even at a fixed shear rate, when the field intensity increases, the magnetic force prevails over the hydrodynamic force, increasing viscosity as the field-induced aggregates grow stronger and resist shear. These findings emphasize the unfavourable influence of polydispersity and higher aggregate size on rheological behaviour and agglomerate formation.

Larger magnetic carriers, as is known, enhance magnetic force on the carriers, boosting targeting efficiency. On the other hand, this increased structure and targeting efficiency may be helpful for various medicinal applications [52].

In our previous investigations [22], to find an explanation for the observed aggregation process, the colloidal interaction among the MNC clusters was investigated. Also, the electrostatic repulsion energy, van der Waals, and magnetic dipole–dipole attraction were calculated in the absence and presence of the external magnetic field to examine the colloidal interaction between the PEG_MNC clusters. Moreover, the field-induced microstructures created with the magnetic field were analysed using an optical microscopic technique.

Based on our findings, we conclude that an external magnetic field induces aggregation, and MNCs are soldered in aggregates, most likely due to bridge contacts among PEG shells or collective engulfment in the PEG of many MNCs.

To demonstrate that the agglomerate generation during targeting is not a particular phenomenon caused by the stent geometry (precisely the local strut arrangement), in this study, during optical microscopy investigations, we evaluated the effect of the two different magnetic field intensities on the structure generation. The morphology and dimensions of the generated agglomerates were compared with the size of the structures found in our earlier investigation (Table 2).

The results in Table 2 demonstrate that even when exposing the suspension to the magnetic field for the same duration, different field intensities generated agglomerates with different shapes and lengths.

For a better understanding of aggregate growth, we display the length history of chain C2 (from Figure 11) over the research period of 30 s.

**Figure 12:**
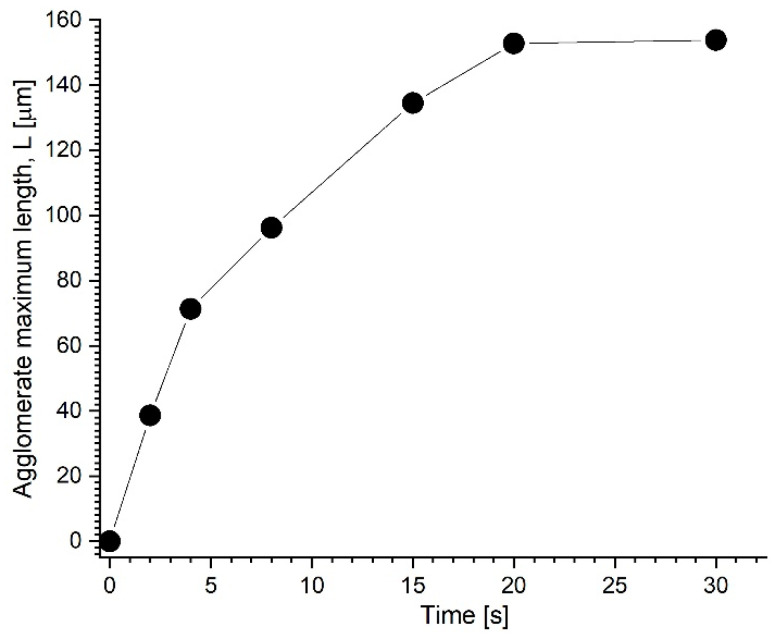
Magnetic-field-induced aggregate maximum length evolution occurred during the investigated period of 30 s. The field intensity is 124 mT, corresponding to the magnet distance from the microscope plate of 7 mm. The experimental L(t) curve corresponds to chain C2 plotted in Figure 11.

Figure 12 depicts the experimental dependence of the maximum aggregate length L on elapsed time t for a sample containing 5% PEG_MNC mass concentration in a magnetic field strength of 124 mT. As presented in the section Effect of the Magnetic Field Intensity, the aggregate length increased with time, a fact confirmed with Figure 12. As the figure shows, the experimental L(t) curves shift in the slope at t = 8 s. Furthermore, the agglomeration process approaches saturation 20 s after applying the magnetic field. After this period, the aggregates’ form and length remain constant. This observed behaviour is strongly connected to the magneto-viscous features described in previous sections.

## 5. Conclusions

According to [38], most ferrofluid systems are stable without a magnetic field. Stability is compromised when exposed to a magnetic field, and the nanoparticles or nanoclusters assemble or agglomerate to regain equilibrium. The consequence was the formation of two separate phases: the carrier fluid and the aggregates.

The processes above were thoroughly examined in the current paper. In this work, we investigated the possible influence of cluster size distribution and polydispersity on agglomerate generations, the correlation between suspension magnetorheological behaviour and agglomerate formations, and the effect of sonication, carrier fluid density, and sedimentation profile on agglomeration processes, in addition to the parameters measured in our previous paper.

Optical microscopy was used to analyse aggregation and agglomeration processes for various magnetic field intensities, and the results related to both the suspension polydispersity index and rheological behaviour.

The size and polydispersity characterization of PEG-coated MNC suspensions using DLS reveals that this suspension contains single clusters and pre-existing aggregates. We hypothesized that MNCs are soldered in aggregates, most likely due to bridge connections between PEG shells or collective engulfment in PEG of numerous MNCs, based on the acquired results and analysis of the PEG_MNC synthesis technique. Furthermore, dispersed particles aggregate or agglomerate, even after ultrasonic treatment, forming sizable structures. This outcome is closely related to the hypothesis previously mentioned.

Rheological studies show that changes in suspension density do not affect the suspension’s rheological and magneto-viscous (MVE) behaviour. According to optical microscopy imaging, only large clusters or pre-existing aggregates produce agglomerates. This assumption is linked to the original polydispersity of the colloid.

The observed MVE behaviour is due to the magnetic component’s multicore nature, resulting in a high induced magnetic moment of particles, favouring their structuring in a magnetic field. The magneto-viscous measurement results are aimed at the viscosity behaviour as a function of time and the rapid formation or destruction of cluster agglomerations when applying or stopping the external magnetic field.

Finally, the generation and development of the chain-like structure are related to the synthesis of PEG-MNC, the colloidal stability of MNC, and the rheological and magnetic properties of the model suspensions.

## Figures and Tables

**Table 1:** Zeta potential and size distribution by Intensity of the PEG-MNCs in water.

Hydrodynamic Diameter, D_H_ (nm)	Zeta Potential (mV)	Polydispersity Index, PDI
4572	−7.22	0.229

**Table 2:** Comparison between the dimensions of the generated agglomerate during optical microscopy in the presence of the different magnetic field intensities.

References	Magnet Type	Dimension (l × w × t) (mm)	Magnet Position Relative to the Microscope Plate (mm)	Field Intensity (mT)	Investigation Period (s)	Maximum Average Length of the Agglomerates (μm)
Early results [22]	NdFeB50	30 × 20 × 20	16	110	5	41
Present investigations	NdFeB52	20 × 10 × 5	7	124	30	146
			12	47	30	69

Where l × w × t = length × width × thickness.

## Data Availability

Data are contained within the article and Supplementary Materials.

## Supplementary Materials

The following supporting information can be downloaded at: https://www.mdpi.com/article/10.3390/pharmaceutics15112612/s1, Video S1: Agglomeration in the presence of the external magnetic field.
